# The accuracy of a smartphone-based hearing assessment compared to conventional audiometry in workers exposed to noise according to sociodemographic, occupational and pre-assessment conditions

**DOI:** 10.1590/2317-1782/e20240260en

**Published:** 2026-07-03

**Authors:** Claire Márcia Santana Lima, Luma Cordeiro Rodrigues Fernandes, Ana Paula Corona

**Affiliations:** 1 Instituto Multidisciplinar de Reabilitação e Saúde, Universidade Federal da Bahia – UFBA - Salvador (BA), Brasil.; 2 Faculdade de Medicina, Universidade Federal da Bahia – UFBA - Salvador (BA), Brasil.; 3 Complexo do Hospital de Clínicas, Empresa Brasileira de Serviços Hospitalares – EBSERH, Universidade Federal do Paraná – UFPR - Curitiba (PR), Brasil.; 4 Departamento de Fonoaudiologia, Faculdade de Medicina, Instituto Multidisciplinar de Reabilitação e Saúde, Universidade Federal da Bahia – UFBA - Salvador (BA), Brasil.

**Keywords:** Workers, Noise-Induced Hearing Loss, Smartphone Application, Accuracy, Related Factors

## Abstract

**Purpose:**

To investigate the sociodemographic and occupational factors, as well as the pre-assessment conditions that influence the accuracy of the hearTest in workers exposed to noise.

**Methods:**

Secondary data from an accuracy study conducted with 232 workers were used. A sociodemographic and occupational questionnaire was administered, and an air conduction hearing assessment of 0.5 to 8 kHz was performed using the hearTest and CA. A Youden index (J) ≥ 70% was considered to define good hearTest accuracy.

**Results:**

When analyzing the identification of any HL by the hearTest, it was observed that sociodemographic factors (age between 40 and 49 years; white race, low education level, and income above five minimum wages) reduced the accuracy of the assessment. When analyzing the identification of NIHL, sociodemographic factors (age between 40 and 49 years; low level of education; and income above two minimum wages) also reduced the accuracy of the assessment, along with the worker's pre-assessment condition (irregular sleep the night before the assessment). For both HL criteria, it was found that the hearTest presented worse accuracy with increasing age. Workers with higher educational levels achieved greater accuracy, and the J was higher among women, workers in technical roles, those aged between 18 and 29 years, non-white, those with higher education, those with an income of two to five minimum wages, and those who denied fasting, for both HL classifications.

**Conclusion:**

The findings indicate that sociodemographic, occupational, and pre-assessment conditions can impact the accuracy of the hearTest assessment in workers.

## INTRODUCTION

Noise-induced hearing loss (NIHL) poses a significant health concern for workers, resulting from prolonged exposure to high sound levels. It burdens the affected population, leading to disability. Globally, occupational noise exposure contributes to an estimated 16% of adult hearing loss (HL) cases, reflecting a substantial impact on over four million disability-adjusted life years (DALYs) annually^(1)^.

The diagnosis of NIHL is carried out using pure tone audiometry, a method considered the gold standard for identifying hearing loss. This procedure must be carried out by a speech therapist in an acoustically treated room or acoustic booth^(2,3)^.

Formal workers subjected to noise levels above 85 decibels have access to periodic audiometric assessments with the aim of monitoring hearing acuity and early detection of the onset or worsening of hearing loss^(4,5)^. Studies show, however, that exposure to noise levels lower than those allowed by legislation can also cause a reduction in hearing thresholds^(6,7)^.

Mobile technologies offer a promising approach for hearing screening, particularly in expanding hearing health programs for noise-exposed workers. These technologies are particularly advantageous for workers in the informal sector or those exposed to noise levels below the thresholds established by legislation^(6,7)^, considering their accessibility and affordability.

Several studies have compared the accuracy of mobile-based tools, including the hearTest smartphone-based hearing assessment device, with conventional audiometry (CA), which is widely recognized as the gold standard. These studies consistently demonstrate the effectiveness of these devices in accurately identifying potential hearing abnormalities^(8,9)^.

The hearTest device provides a convenient alternative for measuring pure tones by air conduction through Android smartphones with calibrated headphones and emit pure tones at frequencies of 0.5, 1, 2, 3, 4, 6, and 8 kHz, with intensity levels ranging from 10 to 90 dBHL^(10)^. It enables users to conduct automated and comprehensive hearing tests in both self-applied and mediated modes, producing conventional audiograms. Despite advancements in mobile-based hearing assessment, more studies still need to investigate the hearTest device's accuracy, specifically among noise-exposed workers^(11)^.

Studies conducted to investigate the accuracy of the hearTest with the population of workers exposed to noise still need to be completed in the literature. The results from Rodrigues et al.^(12)^ revealed good accuracy measures of the device for identifying hearing loss (thresholds above 25dB) in workers, including those with NIHL when compared to CA. However, these studies did not include sociodemographic and occupational data analysis or pre-assessment conditions. In contrast, Pernambuco et al.^(13)^ recommend considering psychological, social, and cognitive factors when evaluating the validity, reliability and diagnostic accuracy measures involved. This guidance is especially relevant when analyzing the accuracy of hearing assessment using subjective methods, such as pure tone audiometry. Additionally, a previous study^(14)^ that investigated the accuracy of self-reported hearing compared to hearing thresholds (HT) obtained through audiometry demonstrated that higher accuracy was associated with younger individuals, non-white individuals, women, and those with higher educational levels.

Therefore, this study aims to investigate the sociodemographic, occupational factors, and pre-assessment conditions that may influence the accuracy of hearing assessment using the hearTest device, compared to CA, among workers exposed to noise.

## METHODS

This study is an exploratory accuracy investigation comparing auditory assessment using the hearTest device with CA. The study utilizes secondary data from a diagnostic accuracy study conducted by Rodrigues et al.^(12)^ in a parastatal company specializing in workplace safety and health management. Therefore, it was decided to describe the sample and procedures of the original study in this session. The sample size was determined based on previous research conducted with adults or the elderly^(15)^, as the target population of noise-exposed workers predominantly consists of adults, and both NIHL and presbycusis primarily affect high frequencies. A sensitivity rate of 76% was assumed, which was the lowest sensitivity identified among the relevant studies^(16)^. The prevalence of NIHL was estimated at 20%^(17)^, and a minimum acceptable confidence limit of 0.5 was set, with a probability of 0.95 that the limit would not be violated. The sample size estimated by Rodrigues et al.^(12)^ was 210 individuals, following the approach proposed by Flahault et al.^(18)^. A margin of 10% was included to anticipate potential non-response, resulting in a final sample size of 231 participants, as determined in the previously mentioned study.

All workers who self-reported noise exposure for any duration or noise exposure level and utilized any form of hearing protection equipment in their work environment were enrolled in the study. Participants who demonstrated a lack of comprehension regarding the auditory assessment procedure with the hearTest device or presented ongoing otorrhea or obstruction of the external acoustic meatus (EAC) were excluded. A lack of comprehension was determined by unsystematic responses during the test. The hearTest app, when identifying an incorrect response rate greater than 20%, signaled through a pop-up on the smartphone screen with a message suggesting that the test be restarted to guarantee reliable answers. Moreover, individuals with mixed or conductive HL in CA were excluded, as these types differ from NIHL, which predominantly manifests as sensorineural hearing loss. The classification of hearing loss type was determined by analyzing the air conduction (AC) thresholds and bone conduction (BC) thresholds obtained during CA.

All participants who attended the aforementioned service between June and September 2018 were required to complete a questionnaire investigating sociodemographic data (gender, age, skin color, education, and monthly income), occupational data (occupation and usual work shift), and pre-assessment conditions (time of the last meal, physical state at the time of assessment using the question "Are you feeling tired?", and sleep condition on the last night using the question "Did you sleep badly last night?"). These factors were investigated as the patient's state of attention is an intrinsic factor that may influence responses during the audiological evaluation^(2)^.

The workers underwent a comprehensive evaluation process, which included an otoscopy examination, hearing assessment with the hearTest device (version 3310), and CA. The otoscopy examination, administration of the questionnaire, and hearTest assessment were conducted by an audiologist affiliated with the research project or a trained undergraduate student in Speech Therapy from a public educational institution in northeastern Brazil. The CA was performed on the same day, immediately following the hearTest assessment, by a professional audiologist independent from the research project and blinded to the hearTest results to minimize potential measurement biases.

The hearTest assessments were conducted using Sennheiser HD280 supra-aural headphones calibrated and connected to a Samsung Galaxy A3 smartphone and was performed using the test-operator response mode, where the examiner presented the sound stimulus and recorded the participant's responses in the application. Participants were instructed to signal by raising their hand each time they heard the sounds, even at low intensity, in a similar way to the research of auditory thresholds in pure tone audiometry. The assessment always began with the left ear, following the device's predetermined configuration. The initial intensity of the test was 40 dBHL for all participants, and the threshold was researched by reducing it to an intensity of 10 in 10 dBHL whenever the test tone was heard and increasing the intensity by 5 dBHL when the test tone was not identified. The application considered the hearing threshold, by frequency, to be the lowest intensity in that there was a positive response in two presentations of the test tone. All the assessments occurred in an occupational health clinic in a soundproof room, although no specific acoustic treatment was applied.

CA was conducted in the same place as the hearTest assessments, using a calibrated audiometer within a controlled acoustic environment. AC thresholds were measured using pure tones at frequencies ranging from 0.25 to 8 kHz. BC thresholds were assessed at frequencies of 0.5, 1, 2, 3, and 4 kHz when necessary.

It is important to note that this study analyzed previously collected data. A descriptive statistical analysis of sociodemographic and occupational characteristics was performed, as well as pre-assessment conditions, to provide a comprehensive characterization of the participant population. Participants were categorized into three occupational groups: (1) industrial, (2) administrative, and (3) technical. Family income was also classified into three strata based on the minimum wage (MW): (1) up to 2 MW (<R$ 2,424.00); (2) 2 to 5 MW (R$ 2,424.00 to R$ 6,060.00); and (3) more than 5 MW (>R$ 6,060.00). Minimum wage is the minimum consideration due and paid directly by the employer to every worker. When the study was carried out the value of the minimum wage was R$ 1,200.00.

The obtained HT from the hearTest and CA were categorized using two criteria. The first criterion classified "normal hearing" when all the HT values were ≤25dBHL in both ears and HL when at least one HT value was more significant than 25dBHL in at least one ear^(12)^. Early detection of potential abnormalities, even if isolated to a single frequency, is crucial for improving workers' protection and safety in their work environment.

The second criterion maintained the definition of "normal hearing" from the first criterion and further categorized the HL into two subgroups: (1) with noise-induced hearing loss (NIHL) configuration and (2) without NIHL configuration. HL with NIHL configuration was identified when at least one ear exhibited CA threshold>25dB in one or more frequencies between 3, 4, and 6kHz, with an improvement observed at the frequency of 8kHz^(12)^. Cases that did not meet the above mentioned descriptions were classified as HL without NIHL configuration.

The sensitivity (Se), specificity (Sp), positive predictive value (PPV), and negative predictive value (NPV) of the hearTest auditory assessment were calculated and compared to those of CA, considering each of the investigated factors. The Youden Index (J) was employed to summarize the accuracy measures, which was computed using the formula: J = Se + Es – 1^(19)^. A cutoff value of J ≥ 70% was established to determine a good accuracy of the hearTest in identifying HL in workers^(12)^.

The present study underwent ethical review and approval by the Ethics and Research Committee of the Institute of Health Sciences at the proposed institution. The study was assigned a Certificate of Presentation of Ethical Appreciation (CPEA) number 55603721.2.0000.5662 and received approval on April 6, 2022, with opinion number 5,333,667. Informed consent was obtained from all participants by signing a Free and Informed Consent Form (FICF).

## RESULTS

A total of 232 workers participated in the study, with the majority being male (94.4%). The participants ranged from 19 to 65 years, with a mean age of 39.2 years (standard deviation [SD] = 11.4). The age group of 30 to 39 had the highest representation, accounting for 34.9% of the participants. Most workers were non-white (94.8%) and had completed or were currently attending high school (66.4%). Regarding family income, 45.2% of the population fell within 2 to 5 times the minimum wage (MW), and 80.2% of the workers were employed in the industrial sector. Regarding the pre-evaluation conditions, only 17.7% of the participants reported experiencing irregular sleep the night before the auditory evaluations, and 18.1% reported feeling tired. Fasting conditions were reported by 73.7% of the participating workers (Table 1).

**Table 1:** Sociodemographic, occupational characterization and pre-assessment conditions to the population of workers exposed to noise (N= 232)

Related Factors	N	%
Sociodemographics		
Gender		
Male	219	94.4
Female	13	5.6
Age Group (years)		
18-29	45	19.4
30-39	81	34.9
40-49	55	23.7
50-65	51	22.0
Educational Background		
≤ Elementary School (<9 years)	41	17.7
High school (9 to <13 years)	154	66.4
≥ University education (13 years or more)	37	15.9
Skin Color		
No-white	220	94.8
White	12	5.2
Income		
< 2 MW	86	37.0
2-5 MW	105	45.2
> 5 MW	41	17.6
Occupational		
Occupation		
Industrial Workers	186	80.2
Technical Workers	39	16.8
Administrative Workers	7	3.0
Work shift		
Night Shift	54	23.3
Day Shift	178	76.7
Pre-assessment conditions		
Irregular Sleep		
Yes	41	17.7
No	191	82.3
Tiredness		
Yes	42	18.1
No	190	81.9
Fasting		
Yes	171	73.7
No	61	26.3

**Caption:** MW= Minimum Wage (1 MW= R$ 1,212.00); up to 2 MW (< 2,424.00); 2-5 MW (2,424.00 to 6,060.00); > 5 MW (> 6,060.00). Source: Research Data

### HearTest accuracy to identify any HL

Table 2 presents the accuracy measures of the hearTest in identifying any HL among workers exposed to noise, as compared to CA, considering various sociodemographic and occupational factors and pre-assessment conditions.

**Table 2:** Accuracy measures of the hearTest smartphone-based device to identify any hearing loss (HL) in noise-exposed workers compared to conventional audiometry (CA) according to sociodemographic, occupational factors and pre-assessment conditions (n= 232)

Accuracy Measures		Sensitivity	Specificity	PPV	NPV	Youden Index
Related factors		% (95% CI)	% (95% CI)	% (95% CI)	% (95% CI)	% (95% CI)
Sociodemographic						
Gender	Male	97.4	77.6	69.8	98.2	75.0
		(90.8-99.7)	(69.9-84.2)	(60.1-78.3)	(93.7-99.8)	(67.3-82.7)
	Female	100.0	81.8	50.00	100.0	81.8
			(48.2-97.7)	(6.8-93.2)		(59.0-104.6)
Age Group (years)	18-29	100.0	90.5	42.9	100.00	90.5
			(77.4-97.3)	(9.9-81.6)		(81.6-99.3)
	30-39	93.3	80.3	51.8	98.1	73.6
		(68.0-99.8)	(68.7-89.1)	(31.9-71.3)	(90.1-99.9)	(57.8-89.5)
	40-49	100.0	51.6	61.5	100.0	51.6
			(33.1-69.8)	(44.6-76.6)		(34.0-69.2)
	≥ 50	97.2	86.7	94.6	92.9	83.9
		(85.5-99.9)	(59.5-98.3)	(81.8-99.3)	(66.1-99.8)	(65.9-101.9)
Skin color	White	100.0	62.5	57.1	100.00	62.5
			(24.5-91.5)	(18.4-90.1)		(28.9-96.0)
	Non-white	97.3	78.8	69.9	98.3	76.1
		(90.6-99.7)	(71.2-85.1)	(60.1-78.5)	(94.0-99.8)	(68.5-83.7)
Educational Background	≤ ElementarySchool	91.7	64.7	78.6	84.6	56.4
	(<9 years)	(73.0-99.0)	(38.3-85.8)	(59.0-91.7)	(54.5-98.1)	(31.1-81.6)
	High school	100.00	79.0	69.0	100.00	79.0
	(9 to <13 years)		(70.0-86.4)	(56.9-79.5)		(71.3-86.8)
	≥ University education	100.00	81.2	45.4	100.00	81.2
	(13 years or more)		(63.6-92.8)	(16.7-76.6)		(67.7-94.8)
Income	< 2 MW	95.6	80.9	64.7	98.1	76.6
		(78.0-99.9)	(69.1-89.7)	(46.5-80.2)	(89.7-99.9)	(63.8-89.4)
	2-5 MW	97.6	79.7	75.5	98.1	77.2
		(87.1-99.9)	(67.8-88.7)	(61.7-86.2)	(89.7-99.9)	(66.3-88.2)
	> 5 MW	100.00	66.7	60.9	100.00	66.7
			(46.0-83.5)	(38.5-80.3)		(48.9-84.4)
Occupational						
Occupation	Industrial Workers	98.4	76.6	67.8	99.0	75.0
		(91.3-100.0)	(68.2-83.7)	(57.1-77.2)	(94.3-100.0)	(66.9-83.1)
	Technical Workers	93.7	82.6	78.9	95.0	76.4
		(69.8-99.8)	(61.2-95.0)	(54.4-93.9)	(75.1-99.9)	(56.8-95.9)
	Administrative Workers	-	85.7	-	100.0	-
			(42.1-99.6)			
Work shift	Day Shift	96.4	76.2	65.1	97.9	72.7
		(87.7-99.6)	(67.7-83.5)	(53.8-75.2)	(92.6-99.7)	(63.7-81.6)
	Night Shift	100.00	84.4	81.5	100.00	84.4
			(67.2-94.7)	(61.9-93.7)		(71.8-97.0)
Pre-Assessment Conditions						
							Irregular Sleep	Yes	100.00	81.8	57.1	100.0	81.8
(64.5-93.0)	(28.9-82.3)	(68.7-95.0)										
No	97.1	76.9	70.8	97.9	74.0		
	(90.1-99.6)	(68.3-84.0)	(60.7-79.7)	(92.6-99.7)	(65.5-82.5)		
Fasting	Yes	96.7	75.4	68.6	97.6	72.2
		(88.6-99.6)	(66.3-83.2)	(57.7-78.2)	(91.8-99.7)	(63.0-81.4)
	No	100.00	84.1	70.8	100.00	84.1
			(69.9-93.4)	(48.9-87.4)		(73.3-94.9)
Tiredness	Yes	93.7	88.5	83.3	95.8	82.2
		(69.8-99.8)	(69.8-97.5)	(58.6-96.4)	(78.9-99.9)	(65.1-99.3)
	No	98.4	75.8	66.3	99.0	74.2
		(91.3-100.0)	(67.4-82.9)	(55.7-75.8)	(94.4-100.0)	(66.1-82.2)

Administrative workers did not present any HL in the assessment with CA

**Caption:** PPV: Positive Predictive Value; NPV: Negative Predictive Value; MW: Minimum Wage

When analyzing the gender variable, the study revealed that both Se and Sp remained consistently high, exceeding 97.4% and 77.6%, respectively, for both male and female participants. However, it was observed that women exhibited slightly higher Se (100%), Sp (81.8%), and NPV measures (100%).

In the analysis of the hearTest performance by age group, it was observed that Se ranged from 93.3% to 100% across all age groups. The Sp varied from 51.6% to 90.5%, with lower values observed in the 40 to 49 age group. NPV consistently remained above 92.9% in all age groups, surpassing PPV, which ranged from 42.9% to 94.6% and achieved higher percentages in workers aged over 50 years.

Concerning skin color, Se and NPV remained consistently above 97.3% and were similar for both groups. However, white workers demonstrated lower Sp and PPV at 62.5% and 57.1%, respectively. Regarding education level, Se, Sp, and NPV increased with higher levels of education, ranging from 64.7% to 100%, considering the three measures previously mentioned. The PPV varied from 45.4% to 78.6% between all strata, but its percentages did not show a linear increase in relation to the level of education.

The income analysis showed that Se was similar among the groups, but this measure increased proportionally in the strata with higher wages, ranging from 95.6% to 100%. Sp was higher in the group with the lowest income (80.9%). NPV remained above 98.1% among all strata, and the PPV was higher among workers with income between 2 and 5 MW (75.5%).

Regarding occupation, Se and NPV remained above 93.7% among all occupations. Se was higher among industrial production workers (98.4%). Sp and NPV ranged from 76.6% to 100% and were higher among workers in the administrative sector. It was not possible to calculate Se, PPV, and J for the group of workers who performed administrative functions since none of them were identified with HL by the CA.

When analyzing the groups according to the work shift, higher Se, Sp, PPV, and NPV values were observed in night shift workers, with measures ranging from 65.1% to 100%. The PPV was the only measure that showed a difference between the groups, with a percentage of 65.1% for day shift workers and 81.5% for those who work night shifts.

Regarding the pre-assessment conditions, workers who reported "irregular sleep" the night before the auditory assessments showed slightly higher Se (100%) and NPV (100%) compared to the other group. However, the PPV was higher (70.8%) for workers who denied "irregular sleep." Regarding the analysis of the "fasting" variable, accuracy measures ranged from 68.6% to 97.6% among workers who reported fasting at the time of assessments. In the other stratum, slightly higher Se, Sp, PPV, NPV, and J measures were observed, ranging from 70.8% to 100%. In the analysis of the variable "tiredness", accuracy measures ranged from 82.2% to 95.8% among workers who reported fatigue and from 66.3% to 99.0% in the other group. Workers who denied feeling tired during the auditory assessments presented higher Se (98.4%) and NPV (99%), while Sp (88.5%) and PPV (83.3%) were higher among those who reported tiredness.

The J, which summarizes the measures of Se and Sp, was estimated as a single numerical value and ranged from 51.6% to 90.5% among all the factors evaluated. The results showed good accuracy (J ≥ 70%) for identifying "any HL" in workers in most of the investigated groups, except for those aged between 40 and 49 years (51.6%), individuals of white ethnicity (62.5%), with schooling up to elementary school (56.4%), and income higher than five MW (66.7%). Higher J values were observed among women (81.8%), workers aged between 18 and 29 years (90.5%), non-white individuals (76.1%), those with higher education (81.2%), income between two and five MW (77.2%), working in technical functions (76.4%), on the night shift (84.4%), and those who reported "irregular sleep" (81.8%), tiredness (82.2%), and denied fasting (84.1%) at the time of auditory evaluations.

### HearTest accuracy for NIHL identification

Table 3 shows the accuracy measures of the hearTest to identify NIHL in workers exposed to noise compared to CA, according to sociodemographic and occupational factors and pre-assessment conditions.

**Table 3:** Accuracy measures of the smartphone-based hearTest device for identifying NIHL in noise-exposed workers compared to conventional audiometry (CA) according to sociodemographic, occupational factors and pre-assessment conditions (N=232)

Accuracy Measures	Sensitivity	Specificity	PPV		NPV	Youden Index
Related factors	% (95% CI)	% (95% CI)	% (95% CI)		% (95% CI)	% (95% CI)
Sociodemographic						
Gender	Male	85.4 (72.2-93.9)	89.5 (83.9-93.6)	69.5 (56.1-80.8)	95.6 (91.2-98.2)	74.9 (63.9-85.9)
	Female*	-	92.3 (64.0-99.8)	-	100.0	-
Age Group (years)	18-29	100.0	95.2 (83.8-99.4)	60.0 (14.7-94.7)	100.0	95.2 (88.8-101.7)
	30-39	80.0 (44.4-97.5)	91.5 (82.5-96.8)	57.1 (28.9-82.3)	97.0 (89.6-99.6)	71.5 (45.9-97.2)
	40-49	88.2 (63.6-98.5)	76.3 (59.8-88.6)	62.5 (40.6-81.2)	93.5 (78.6-99.2)	64.5 (44.1-85.0)
	≥ 50	83.3 (58.6-96.4)	93.9 (79.8-99.3)	88.2 (63.6-98.5)	91.2 (76.3-98.1)	77.3 (58.2-96.3)
Skin color	White	100.0	70.0 (34.7-93.3)	40.0 (5.3-85.3)	100.0	70.0 (41.6-98.4)
	Non-white	84.8 (71.1-93.7)	90.8 (85.5-94.6)	70.9 (57.1-82.4)	95.8 (91.4-98.3)	75.6 (64.3-86.8)
Educational Background	≤ Elementary School (<9 years)	81.2 (54.3-95.9)	80.0 (59.3-93.2)	72.2 (46.5-90.3)	87.0 (66.4-97.2)	61.2 (36.5-86.0)
	High school (9 to <13 years)	86.2 (68.3-96.1)	92.0 (85.8-96.1)	71.4 (53.7-85.4)	96.6 (91.6-99.1)	78.2 (64.8-91.6)
	≥ University education (13 years or more)	100.0	88.2 (72.5-96.7)	42.9 (9.9-81.6)	100.0	88.2 (77.4-99.1)
Income	< 2 MW	76.9 (46.2-95.0)	90.4 (81.2-96.1)	58.8 (32.9-81.6)	95.6 (87.8-99.1)	67.3 (43.5-91.2)
	2-5 MW	89.3 (71.8-97.7)	89.6 (80.5-95.4)	75.8 (57.7-88.9)	95.8 (88.3-99.1)	78.9 (65.6-92.2)
	> 5 MW	85.7 (42.1-99.6)	88.2 (72.5-96.7)	60.0 (26.2-87.8)	96.8 (83.3-99.9)	73.9 (45.9-102.0)
Occupational						
Occupation	Industrial Workers	83.8 (68.0-93.8)	89.3 (83.1-93.7)	66.0 (50.7-79.1)	95.7 (90.8-98.4)	73.0 (60.2-85.9)
	Technical Workers	90.9 (58.7-99.8)	92.9 (76.5-99.1)	83.3 (51.6-97.9)	96.3 (81.0-99.9)	83.8 (64.3-103.2)
	Administrative Workers	-	85.7 (42.1-99.6)	-	100.0	-
Work shift	Day Shift	87.2 (72.6-95.7)	88.5 (82.0-93.3)	68.0 (53.3-80.5)	96.1 (91.1-98.7)	75.7 (63.9-87.4)
	Night Shift	77.8 (40.0-97.2)	93.3 (81.7-98.6)	70.0 (34.7-93.3)	95.4 (84.5-99.4)	71.1 (43.0-99.2)
Pre-Assessment Conditions						
Irregular Sleep	Yes	83.3 (35.9-99.6)	82.9 (66.3-93.4)	45.4 (16.7-76.6)	96.7 (82.8-99.9)	66.2 (33.9-98.5)
	No	85.7 (71.5-94.6)	91.3 (85.5-95.3)	73.5 (58.9-85.0)	95.8 (91.0-98.4)	77.0 (65.5-88.5)
Fasting	Yes	86.5 (71.2-95.5)	88.1 (81.3-93.0)	66.7 (51.6-79.6)	95.9 (90.8-98.7)	74.5 (62.2-86.8)
	No	81.8 (48.2-97.7)	94.0 (83.4-98.7)	75.0 (42.8-94.5)	95.9 (86.0-99.5)	75.8 (52.1-99.5)
Tiredness	Yes	76.9 (46.2-95.0)	93.1 (77.2-99.1)	83.3 (51.6-97.9)	90.0 (73.5-97.9)	70.0 (45.3-94.7)
	No	88.6 (73.3-96.8)	89.0 (83.0-93.5)	64.6 (49.5-77.8)	97.2 (92.9-99.2)	77.6 (66.0-89.2)

Female participants did not present NIHL in the assessment conducted with CA

**Caption:** PPV: Positive Predictive Value; NPV: Negative Predictive Value; MW: Minimum Wage.

**Source:** Research data

Regarding gender, women exhibited higher Sp (92.3%) and NPV (100%) measures. However, Se, PPV, and J could not be estimated for women, as none of them were identified with HL by the CA.

In the analysis of the hearTest performance by age group, it was observed that Se, Sp, and NPV remained above 76.3% for all age groups. However, the Sp and NPV showed a reduction in their percentages with the increase in age groups, except for Sp in workers aged over 50 years (93.9%). This relationship was not observed in the other investigated accuracy measures. The PPV presented percentages ranging from 57.1% to 88.2%, with the highest percentage obtained among workers aged over 50 years (88.2%).

When considering skin color, it was observed that non-white workers had higher Sp at 90.8% and PPV at 70.9%. Among white workers, Se and NPV showed 100% correspondence between the results obtained with hearTest and CA. Regarding education, Se (81.2% to 100%) and NPV (87% to 100%) showed higher percentages for workers with a higher educational level. However, the PPV showed an inverse relationship, with a percentage of 42.9% of agreement between the tests among workers with higher education and 72.2% among those with a lower level of education.

In the income analysis, Se was higher (89.3%) in the group with a salary of 2 to 5 MW, while Sp was higher among those with an income below 2 MW (90.4%). The PPV ranged from 58.8% to 75.8%, and the NPV from 95.6% to 96.8%.

Regarding occupation, technical workers showed higher Se (90.9%), Sp (92.9%), and PPV (83.3%), while those with an administrative function had a higher NPV (100%). However, it was not possible to calculate Se, PPV, and J for the group of workers with an administrative function, as none of them were identified with NIHL by the CA.

In the analysis by work shift, Se was higher (87.2%) in day shift workers, while Sp was higher in night shift workers (93.3%). The PPV and NPV were similar between groups, ranging from 68% to 96.1%.

Regarding the pre-assessment conditions, workers who reported sleeping well had higher Se, Sp, and PPV (85.7%, 91.3%, and 73.5%, respectively). NPV was high and similar for both groups (96.7% and 95.8%)**.** Considering the "fasting" variable, Sp (94%) and PPV (75%) were higher among those who were fed at the time of the assessments. Se was higher in the group that performed the assessments in a fasting state, and NPV was the same between the groups**.** For the variable "tiredness”, "Se (88.6%) and NPV (97.2%) were slightly higher among workers who denied being tired at the time of the assessments. Sp (93.1%) and PPV (83.3%) were higher among those who reported tiredness during the tests.

The J ranged from 61.2% to 95.2% among all evaluated factors, showing good accuracy (J ≥ 70%) for identifying NIHL in workers in most of the strata evaluated, except for those aged between 40 and 49 years (64.5%), with schooling up to elementary school (61.2%), income below 2 MW (67.3%), and those who reported irregular sleep (66.2%) the night before the auditory assessment. Additionally, J was higher among workers aged 18 to 29 years (95.2%), non-white (75.6%), with higher education (88.2%), income of two to five MW (78.9%), working in technical positions (83.8%), on a day shift (75.7%), and those who denied "irregular sleep" (77.0%), tiredness (77.6%), and fasting at the time of hearing evaluations (75.8%).

## DISCUSSION

Considering the criteria adopted by this study, the hearTest demonstrated good accuracy (J ≥70%) for identifying "any HL" in all evaluated strata, except for workers aged between 40 and 49 years, white individuals, those with low education, and income above five MW. For NIHL detection, the hearTest showed suboptimal accuracy in the same age and education groups, as well as for those with income less than two MW and those who reported irregular sleep the night before the hearing assessment. Notably, workers aged over 50 years exhibited better accuracy. Higher educational levels were associated with better accuracy in both "any HL" and NIHL identification using hearTest. Interestingly, the device showed greater accuracy among individuals with lower income when detecting "any HL", but no such relationship was observed for NIHL. Moreover, women, workers aged between 18 and 29 years, non-white individuals, those with higher education, income ranging from two to five MW, and those in technical occupations displayed higher accuracy measures (J) in both "any HL" and NIHL classifications using hearTest. These individuals also denied fasting during the auditory evaluations, contributing to improved accuracy.

The superior accuracy of hearTest in identifying HL among women compared to men should be interpreted with caution, as the number of female participants in the sample was limited. Nevertheless, it is well-established that women tend to exhibit a higher awareness of healthcare and a more robust culture of preventive measures^(20)^. These findings are consistent with the results reported by Kamil et al.^(14)^.

Regarding age, the findings from our study align with those of Rueda and Monteiro^(21)^, who observed higher average scores in adults aged 18 to 25 when evaluating attention capacity using a test. This skill is crucial for detecting stimuli during auditory assessments.

A previous study^(22)^ reported superior performance in auditory assessments among participants with higher incomes. The authors associated these findings with better access to health and education-related information for higher-income individuals. However, the current study did not observe a similar correlation. In the present study, the finding of lower accuracy of the hearTest among higher-income workers for identifying any hearing loss was attributed to less familiarity with the test. This can be explained by the fact that, in general, these workers have a higher educational level and occupy roles with less noise exposure^(23)^, which results in a lower frequency of audiometry tests. The hearTest demonstrated differential accuracy in identifying "any HL" and NIHL depending on specific factors. Workers on night shifts and those who reported "irregular sleep" and tiredness during assessments showed better hearTest accuracy for detecting "any HL". This initially unexpected finding can be explained by a compensatory response of the central nervous system, characterized by the additional recruitment of specific resources directly related to the cognitive demands of the task. This recruitment reflects an adaptive effort by the brain to maintain performance in the face of challenges imposed by sleep deprivation and other unfavorable conditions^(24-26)^. Additionally, studies suggest that greater cognitive reserve capacity may make some individuals less susceptible to sleep deprivation^(26)^. Furthermore, considering that the audiological assessment was performed in a non-acoustically treated environment and that, although the hearTest software performs a prior noise check before measuring hearing thresholds, it is plausible to assume that environmental noise may have compromised the detection of stimuli in low and mid frequencies. Thus, hearing thresholds in these frequencies may be worse, indicating "any hearing loss." This effect, however, would not impact the identification of noise-induced hearing loss (NIHL), since this condition is characterized by higher hearing thresholds at high frequencies. On the other hand, for identifying NIHL, the hearTest performed better in workers on day shifts and those who denied "irregular sleep" and tiredness during tests. It is essential to consider that, in addition to the aforementioned factors, these results may be influenced by various unexamined factors, such as participants' prior experience with hearing tests, the presence of tinnitus, HL severity, and other multifactorial aspects involved in hearing assessments.

In the study conducted by Rodrigues et al.^(12)^, the accuracy measures of the hearTest to identify "any HL" in workers showed a range of J from 33.0% to 77.7%, with Se ranging from 29.6% to 91.6% and Sp from 83.9% to 99.4%. In contrast, using the same definition of HL, our present study found a broader range of J, from 51.6% to 90.5%, with Se ranging from 91.7% to 100% and Sp from 51.6% to 90.5%. When investigating the accuracy of the hearTest for identifying NIHL in workers, the previous study^(20)^ reported a range of J from 29% to 75.5%, Se from 29.6% to 91.6%, and Sp from 83.9% to 99.4%. In our present study, the accuracy (J) of the hearTest for identifying NIHL showed a wider range, from 61.2% to 95.2%, with Se and Sp varying between 76.9% and 100% and from 70% to 95.2%, respectively. The NPV remained consistently similar and above 79.7% in both studies. However, the PPV showed lower but comparable percentages, ranging from 54.6% to 95.6% in the previous study and 40.0% to 94.6% in the present study, for all HL strata and classifications. The observed differences between the results of the studies can be attributed to methodological dissimilarities, such as the individual analysis of results and the inclusion of accuracy analysis based on sociodemographic and occupational factors and pre-assessment conditions.

In a previous study by Corona et al.^(27)^, which evaluated the accuracy of the hearTest in identifying "some degree of HL" in adults aged over 15, they reported Se and NPV measurements greater than 90%, consistent with our present study's findings. However, their study showed Sp and PPV values above 75.5% for the same population and HL criterion and a J range of 75.1% to 79.1%. In contrast, our present study observed Sp ranging from 51.6% to 90.5%, PPV from 42.9% to 94.6%, and J from 51.6% to 90.6%. These discrepancies maybe attributed to the differing criteria used for classifying HL. While we classified any HT >25dBHL as any HL, Corona et al.^(27)^ considered the mean of 0.5, 1, 2, and 4kHz >25dBHL for the same HL classification. Additionally, the age range of the studied population also varied, with their study encompassing adults aged 15 to 92 years, while our present study focused on individuals aged 19 to 65 years. These methodological distinctions likely contribute to the variations in the study outcomes.

The findings of Kamil et al.^(14)^ regarding age, education, and skin color align with the results obtained in our study, supporting higher accuracy in the younger population and reduced accuracy measures in individuals with lower education levels. Moreover, we also observed higher accuracy among non-white workers for both HL classifications, while skin color influenced accuracy only among white workers (J = 62.5%). It is important to emphasize that the analysis of the hearTest's accuracy according to the variable "skin color" must take into account other factors such as income and education, as these define the functions occupied and, consequently, greater or lesser noise exposure at work. A study that investigated the prevalence of occupational noise exposure in Brazilian workers found that a higher prevalence of occupational noise exposure was associated with workers with a high school education and black race/skin color. Thus, it is possible to assume that the lesser experience of white workers with the test, given their occupation of functions with less noise exposure, may have influenced the determination of hearing thresholds. The learning effect of the test cannot be ruled out, with a possible improvement in the accuracy of responses to auditory stimuli, identified among workers exposed to high noise levels and who undergo periodic annual or biannual sequential audiometry^(28)^ more frequently, when compared to workers exposed to less intense noise levels or intermittent exposure. It is crucial to note that the methodological design used in their study differs from ours, as they employed association measures for the statistical analysis to estimate the precision (accuracy) of the self-reported auditory assessment. Additionally, their study focused on an age group over 50 years, and the HL criterion used (average of air HT greater than 25dBHL in the frequencies of 0.5, 1, 2, and 4kHz) also varied from our present study. Moreover, the hearing assessment instrument utilized by Kamil et al.^(14)^ was self-reported, which might be less accurate than the hearTest, the device used in our study. Factors such as age and gender can significantly influence self-perception of hearing. Conversely, the hearTest, being quite similar to the CA, is capable of obtaining auditory responses closer to the gold standard, making it more suitable for research and clinical purposes in hearing health programs and public health policies, despite its higher cost compared to self-report auditory responses. Additionally, it is noteworthy that Kamil et al.^(14)^ did not consider income factors, occupational variables, or pre-assessment conditions, which we investigated in our present study.

The results of our study are highly relevant, as it is the first to investigate the impact of sociodemographic and occupational factors, as well as pre-assessment conditions, on the accuracy of hearing assessment using the hearTest in workers exposed to noise. These findings hold the potential to contribute significantly to the management of intrinsic and extrinsic factors that may influence the accuracy of this tool in the worker population. For instance, providing prior guidance and necessary preparation for the evaluation, as well as optimizing the methodology and instructions during the test, can enhance the accuracy of the hearTest. Utilizing this device can expand the availability of hearing health programs, particularly targeting at-risk groups for occupational hearing loss that are currently not covered by existing public hearing health policies. Furthermore, the data obtained in this study can be valuable in population surveys to estimate the prevalence of auditory pathologies, identify priority groups for intervention, and support effective public health measures.

One limitation of the present study was the predominant composition of the sample, consisting mainly of non-white male workers in the industrial production sector, as well as the unequal distribution of participants among the analyzed strata. The gender and occupational function distribution also reflected the companies’ profile, mainly concentrated in civil construction and industrial production, where male workers were more prevalent. Regarding race, it is essential to note that the study was conducted in Salvador, a municipality with a population composed of approximately 82.1% black individuals, in the state of Bahia, where 80.2% of the population is non-white^(29)^. Additionally, the heterogeneous number of participants in the investigated strata and the separate analysis of variables of interest without considering multifactorial factors that could influence the perception of auditory pathologies and accuracy during evaluation may have impacted the results. Also, this study wasn’t considering the tests counterbalancing. Interchange tests would also have minimized any potential order effects and potential bias like fatigue, concentration, and tiredness. Conducting the tests in different testing environments could have limited the true examine the effects of sociodemographic, occupational, and pre-assessment characteristics. However, previous studies carried out in non-clinical environments have already identified good accuracy of hearTest for identifying hearing loss even in these environments, and this strategy was a methodological option to consider informal workers who would not have access to clinical environments. Thus, to address these limitations, conducting new studies with more homogeneous groups, controlling the sample size and interchange tests is recommended. Such efforts will contribute to enhancing the robustness and generalizability of the findings.

## CONCLUSION

The findings of this study indicate that sociodemographic and occupational factors, as well as pre-assessment conditions, can impact the accuracy of hearing assessments with the hearTest device in noise-exposed workers, particularly age, race, education level, and sleep patterns.
